# A preliminary exploration of the role and mechanisms of CD93 in promoting the malignant progression of head and neck squamous cell carcinoma

**DOI:** 10.3389/fphar.2026.1847632

**Published:** 2026-07-08

**Authors:** Jing Zhou, Yao Zhang, Nian Zhang, Yan Liao, Li Guo, Deqin Yang

**Affiliations:** 1 College of Stomatology, Chongqing Medical University, Chongqing, China; 2 Chongqing Key Laboratory of Oral Diseases, Chongqing, China; 3 Chongqing Municipal Key Laboratory of Oral Biomedical Engineering of Higher Education, Chongqing, China; 4 Chongqing Municipal Health Commission Key Laboratory of Oral Biomedical Engineering, Chongqing, China; 5 Department of Stomatology, The First Affiliated Hospital of Chengdu Medical College, Chengdu, China; 6 Department of Conservative Dentistry and Endodontics, Shanghai Stomatological Hospital and School of Stomatology, Fudan University, Shanghai, China; 7 Shanghai Key Laboratory of Craniomaxillofacial Development and Diseases, Fudan University, Shanghai, China

**Keywords:** angiogenesis, CD93, HNSCC, TAM, Wnt/β-catenin

## Abstract

**Background:**

Head and neck squamous cell carcinoma (HNSCC) is a highly invasive cancer with an immunosuppressive microenvironment. Although CD93 promotes angiogenesis in tumor endothelial cells, its role in HNSCC tumor cells and the impact of CD93-mediated regulation of tumor cells on the tumor microenvironment are unclear. This study investigates whether CD93 promotes the malignant progression of HNSCC by enhancing tumor cell malignancy and contributing to macrophage-associated and angiogenic remodeling of the tumor microenvironment, elucidating the underlying mechanisms.

**Methods:**

Bioinformatics analyses assessed CD93 expression, clinical relevance, immune infiltration, and signaling pathways in HNSCC. CD93 expression was validated in clinical specimens and cell lines. CD93 knockdown and overexpression models were used to examine invasion, migration, proliferation, and epithelial–mesenchymal transition (EMT). Conditioned media from CD93-modulated tumor cells were applied to THP-1-derived macrophages and HUVECs to assess macrophage-associated markers and endothelial tube formation. Wnt/β-catenin signaling was pharmacologically activated or inhibited. Xenograft growth and macrophage depletion were evaluated in BALB/c nude mice. Protein–protein docking was used to explore predicted spatial compatibility between CD93 and receptor-proximal Wnt pathway components.

**Results:**

CD93 was upregulated in HNSCC and associated with poor prognosis and an immunosuppressive, pro-angiogenic TME. CD93 knockdown inhibited invasion, migration, proliferation, EMT-associated changes, CD206 expression, changes in macrophage-associated markers, and endothelial tube formation, whereas CD93 overexpression produced opposite effects. CD93 expression was associated with Wnt/β-catenin activation; CHIR99021 reversed the effects of CD93 knockdown, whereas XAV939 attenuated changes induced by CD93 overexpression. *In vivo*, CD93 knockdown suppressed xenograft growth, proliferation, angiogenesis, EMT-associated changes, CD206-positive macrophage-associated signals, and β-catenin expression. Clodronate liposome-mediated macrophage depletion reduced HSC3-shNC tumor growth and narrowed the difference between HSC3-shNC and HSC3-shCD93 tumors. Docking analysis indicated that CD93 showed the most favorable predicted spatial compatibility with LRP6 E1E2 among the tested Wnt receptor-related components.

**Conclusion:**

CD93 may promote HNSCC progression by enhancing tumor-cell aggressiveness and tumor microenvironment remodeling, at least partly through Wnt/β-catenin-related signaling. Docking analysis provides a preliminary structural hypothesis for a potential CD93–LRP6 E1E2 spatial association. CD93 may represent a prognostic biomarker and candidate therapeutic target in HNSCC.

## 1 Introduction

Head and neck squamous cell carcinoma (HNSCC) is one of the most prevalent malignant tumors, with substantial morbidity and mortality rates (Barsouk et al., 2023; Sung et al., 2021). In recent years, surgery, chemoradiotherapy, EGFR-targeted therapy, and immune checkpoint inhibitors have been incorporated into standard treatment; however, the overall 5-year survival rate has improved only modestly. Local invasion, lymphatic metastasis, therapeutic resistance, and immune escape continue to limit clinical outcomes (Sung et al., 2021). Therefore, a deeper molecular understanding of HNSCC progression and the identification of biologically and therapeutically meaningful targets remain urgently needed.

Evidence shows that HNSCC progression depends not only on tumor cells but also on the tumor microenvironment (TME) (Meng et al., 2024). The TME comprises tumor-associated vascular endothelial cells, fibroblasts, and diverse immune cells, among which tumors-associated macrophages (TAMs) play a critical role (Meng et al., 2024; Troiano et al., 2019). Reviews and meta-analyses suggest that in HNSCC, TAM infiltration and an M2-like phenotype are related to advanced clinical stage, increased invasiveness, and adverse prognosis, indicating that macrophage functional states are closely linked to HNSCC progression (Troiano et al., 2019; Snietura et al., 2020; He et al., 2024; Fu et al., 2020). This raises the question of whether there are molecules in HNSCC that both shape a macrophage-associated microenvironment and directly promote malignant behavior in HNSCC.

CD93 is a highly glycosylated transmembrane protein. Previous studies focused primarily on its role in endothelial cell adhesion, migration, and angiogenesis (Tossetta et al., 2023; Guo et al., 2026). Animal studies have shown that CD93 regulates tumor vascular structure and function, and that blocking CD93-related signaling can improve vascular maturity, increase perfusion, and enhance the delivery of chemotherapeutic drugs and immune cells, thereby boosting antitumor efficacy (Sun et al., 2021). In addition, multiple pan-cancer analyses based on public databases such as TCGA have revealed that CD93 is abnormally expressed in various tumors, significantly associated with the infiltration of various immune and stromal cell populations (Guo et al., 2022; Tong et al., 2021; Zhang et al., 2022).

Taken together, CD93 in HNSCC may be more than a vascular- or immune-related marker; it may also function as a driver of disease progression. Although existing pan-cancer studies suggest that CD93 correlates with M2 macrophage infiltration and pathways such as Wnt and epithelial-mesenchymal transition (EMT), these findings remain largely based on bioinformatics analysis (Guo et al., 2022; Tong et al., 2021; Zhang et al., 2022). No studies have directly demonstrated whether CD93 promotes malignant behavior in tumor cells and, through these cells, further alters macrophage polarization and promotes angiogenesis to drive the malignant progression of HNSCC. Therefore, we hypothesize that CD93 is upregulated in HNSCC, promotes the malignant behavior of tumor cells, and, through tumor cell-derived effects, contributes to macrophage-associated and angiogenic remodeling of the TME, ultimately accelerating HNSCC progression.

To test this hypothesis, we first analyzed publicly available HNSCC datasets and validated CD93 expression in clinical specimens and cell lines. We then examined the effects of CD93 knockdown or overexpression on malignant tumor cell phenotypes, macrophage-associated marker profiles, and endothelial tube formation. To investigate the mechanism, we combined CD93 modulation with pharmacological activation or inhibition of Wnt/β-catenin signaling. We further assessed CD93-dependent tumor growth in xenograft models and performed macrophage depletion using clodronate liposomes to evaluate the functional contribution of macrophages *in vivo*. Finally, protein–protein docking was used to explore whether the CD93 extracellular domain showed predicted spatial compatibility with receptor-proximal Wnt pathway components.

## 2 Materials and methods

The overall experimental workflow is summarized in Supplementary Figure S0.

### 2.1 Public database analysis

Analyses of CD93 expression, survival, pathological, macrophage subtypes, immune infiltration, and signaling-pathway enrichment were performed using the TCGA and GEO databases via the BEST platform (Liu et al., 2023) and TIMER 3.0 (Cui et al., 2025).

### 2.2 Clinical specimens

Fifty HNSCC tissue specimens (n = 50) and six adjacent normal tissue specimens (n = 6) were obtained from commercially purchased HNSCC tissue microarrays supplied by Shanghai Xinchao Biotechnology Co., Ltd. Ethical approval for the use of these human tissue specimens was granted by the Ethics Committee of Shanghai Xinchao Biotechnology Co., Ltd. (approval no. SHYJS-CP-230801).

### 2.3 Cell culture

The HNSCC cell lines HSC3, CAL27, and HN6, the normal oral keratinizing epithelial cell line HOK, together with human umbilical vein endothelial cells (HUVECs), were obtained from Shanghai Zhongqiao Xinzhou Biotechnology Co., Ltd., and authenticated by colleagues (Yang et al., 2024; Liao et al., 2024). HUVECs were cultured in endothelial cell medium (ECM, ScienCell, United States). THP-1 cells (CL-0233) were provided by Wuhan Punoise Life Technology Co., Ltd. The Wnt pathway inhibitor XAV939 was used at a concentration of 20 μM (Acharya et al., 2024), and the activator CHIR99021 at 6 μM (Lin et al., 2018). Tumor and epithelial cells were cultured in DMEM medium, HUVECs in ECM medium, and THP-1 cells in RPMI-1640 medium. All of the above media were supplemented with 10% fetal bovine serum. All cell manipulations were performed under sterile conditions in a laminar-flow hood, and cells were cultured at 37 °C in 5% CO_2_. All cell lines were routinely tested for *mycoplasma* contamination and confirmed to be negative before use in experiments.

### 2.4 Cell transfection

Two shRNAs targeting human CD93 were designed based on the human CD93 transcript (NM_012072.4). The target sequences were as follows: shCD93-1, 5′-GCC​TTA​CTC​TAA​CTG​GCA​CAA-3′; shCD93-2, 5′-CGA​GAC​TCA​GAG​TCA​TTA​TTT-3′. A non-targeting shRNA served as the negative control during lentiviral transduction in HSC3 and CAL27 cells. Stable cell lines were then established through puromycin selection at a concentration of 1.2 μg/mL. Validation of CD93 protein expression was conducted via Western blot analysis.

Stable CD93-overexpressing cell lines were established using the pLV-ZsGreen (2A)PURO-CMV lentiviral vector carrying the GFP reporter gene. Lentiviral supernatant containing pLV-ZsGreen (2A)PURO-CMV-CD93 (with empty vector as control) was added, and cells were infected in complete medium containing 10 μg/mL polybrene. GFP signals were observed under a fluorescence microscope to assess transduction efficiency. Stably transduced cells were selected with 1.2 μg/mL puromycin. Validation of CD93 protein expression was done through Western blot.

#### 2.4.1 Preparation of tumor cell culture supernatant

Transfected HSC3 and CAL27 cells were seeded in 100 mm culture dishes and grown in complete DMEM medium with 10% FBS. Upon reaching 70%–80% confluence, cells were rinsed with PBS without antibiotics and incubated for 24 h in low-serum DMEM supplemented with 2% FBS. The supernatant was collected, centrifuged at 1,500 rpm for 10 min to remove cells and debris, and filtered through a 0.22-μm filter. The tumor cell culture supernatant obtained was divided into portions and preserved at either 4 °C for immediate use or at −80 °C for long-term storage. For the blank control supernatant, an equal volume of low-serum medium was incubated in HOK culture flasks under identical conditions for 24 h. The supernatant was centrifuged, filtered, and stored as described.

#### 2.4.2 Effect of tumor cell supernatant on THP-1 cells

##### 2.4.2.1 Maintenance and induction of differentiation in THP-1 cells

THP-1 cells were seeded at 5 × 10^5^ cells/mL density in a 6-well plate with 2 mL per well. Phorbol 12-myristate 13-acetate (PMA) at a concentration of 100 ng/mL was used to stimulate adhesion and differentiation for 24 h. After removing the PMA-containing medium, fresh complete PMA medium was supplied. The cells were then incubated for an additional 24 h to establish a stable population of adherent macrophage-like THP-1 cells.

##### 2.4.2.2 THP-1-derived macrophages were treated with tumor cell supernatant

Treatment solutions were applied based on the assigned experimental groups. In the tumor cell supernatant group, culture supernatants from shCD93 or shNC-treated HSC3 and CAL27 cells were combined in a 1:1 ratio with RPMI-1640 supplemented with 2% FBS. For the blank-control group, the pre-prepared blank control supernatant was mixed 1:1 with RPMI-1640, supplemented with 2% FBS.

### 2.5 Immunohistochemistry

Immunohistochemistry was performed following a previously published protocol (Liao et al., 2021). The antibodies used were CD93 (Proteintech, 18283-1-AP, 1:500), CD31 (Cell Signaling, 3528T, 1:1000), N-cadherin (Cell Signaling, 13116T, 1:200), E-cadherin (Cell Signaling, 24E10, 1:1,000), CD86 (Cell Signaling, 91882T, 1:200), CD206 (Immunoway, YM8349, 1:200), F4/80 (Cell Signaling, 70076T, 1:200), Ki-67 (Zenbio, P46013, 1:50), and β-catenin (Zenbio, R23616, 1:200). The immunohistochemical staining intensity was evaluated following established criteria (Wang et al., 2021). IHC staining was independently quantified by two investigators blinded to the experimental groups, and the average value was used for statistical analysis.

### 2.6 RT-qPCR

The method was as previously described (Guo et al., 2025). Primer sequences were as follows:

**Table:** 

CD93-F	TGT​GGA​TGA​GTG​TGC​TCT​GG
CD93-R	GGA​CCC​TTG​TGT​GTT​GAA​GC
GAPDH-F	GCA​CCG​TCA​AGG​CTG​AGA​AC
GAPDH-R	TGG​TGA​AGA​CGC​CAG​TGG​A

### 2.7 Western blot analysis

Protein extracted from cells was quantified and an equivalent amount was separated on an SDS-PAGE gel, then transferred to a PVDF membrane. The membrane was blocked with 5% skimmed milk powder in TBST for 1.5 h. Next, it was incubated overnight at 4 °C with primary antibodies: CD93 (Invitrogen, PA5-52664, 1:1,000), E-cadherin (Cell Signaling, 24E10, 1:1,000), N-cadherin (Cell Signaling, D4R1H, 1:1,000), ZO-1 (Cell Signaling, D7D12, 1:1,000), β-actin (Cell Signaling,13E5, 1:1,000), CD86 (Immunoway, YT7823, 1:1,000), CD206 (Immunoway, YM834, 1:1,000), iNOS (Cell Signaling, 13120, 1:1,000), TNF-α (Cell Signaling, 11948, 1:1,000), IL-6 (Immunoway, YM8783, 1:1,000), β-catenin (Zenbio, R23616, 1:1,000), Cyclin D1 (Zenbio, R380999, 1:1,000), and c-Myc (Zenbio, R380784, 1:1,000). Subsequently, the membranes were incubated with secondary antibody (Immunoway, RS0011, 1:10,000) for 1.5 h. Protein bands were visualized using a chemiluminescent substrate (P0018FS, BeyoECL Moon) and quantified with ImageJ. The relative expression level of each target protein was calculated as the intensity of the target protein band divided by the intensity of the corresponding internal control band from the same sample. The resulting relative expression values were used for statistical analysis.

### 2.8 Transwell

Cell invasion was evaluated by employing Transwell chambers precoated with Matrigel. Initially, 100 μL of matrigel was evenly distributed in the upper chamber, followed by seeding of cells. Following a 24-h incubation period, cells were fixed using methanol and stained with 0.1% crystal violet. Subsequently, cells present on the upper side of the membrane were delicately eliminated, and images of the lower membrane surface were captured using a microscope.

### 2.9 Wound-healing assay

Cells were plated in 6-well dishes and grown until they reached 90% confluence. A linear wound was created with a sterile 200-μL pipette tip. Subsequently, cells were cultured in medium devoid of serum. Photographs were taken at 0, 12, and 24 h using a light microscope.

### 2.10 EdU assay

Cell proliferation was assessed via the kFluor488 Click-iT EdU Kit (KeyGen). The specific operation is as described previously (Guo et al., 2025). Images were acquired randomly.

### 2.11 Tube formation assay

A total of 150 μL of prechilled Matrigel was evenly dispensed into each well of a 24-well plate and allowed to solidify at 37 °C for 30 min. HUVECs were then seeded onto the solidified Matrigel after treatment with tumor cell supernatant or blank control supernatant. The corresponding supernatants, mixed at a 1:1 (v/v) ratio with ECM containing 2% FBS, were added to each well. Following an 8-h incubation at 37 °C, viable cells were stained with Calcein Green CM (Beyotime) at a 1:1000 dilution for 30 min before imaging. Images of tubular structures were captured using a Leica microscope and analyzed using ImageJ (Liao et al., 2024).

### 2.12 Immunofluorescence

Cells were treated with 4% paraformaldehyde for 15 min at room temperature, then permeabilized using PBS with 0.1% Triton X-100 for 10 min. Subsequently, a blocking step with 5% goat serum at room temperature for 1 h was performed, followed by overnight incubation at 4 °C with anti-β-catenin antibody (Zenbio, R23616, 1:500). After washing, cells were exposed to Alexa488-conjugated secondary antibody (1:500, A12924, Thermo Fisher Scientific) at room temperature for 1 h. Nuclei were stained with DAPI (C1006, BiYunTian) for 5 min. Imaging was conducted using a confocal microscope (SP8, Leica, Germany) and analyzed utilizing ImageJ.

### 2.13 Establishment of subcutaneous tumor model in animals

Male BALB/c nude mice were maintained in specific pathogen-free conditions for *in vivo* xenograft experiments. HSC3 cells expressing shNC or shCD93 were injected subcutaneously to establish xenograft tumors. Tumor size was monitored regularly, and volume was calculated using the formula length × width^2^/2. After 2 weeks, mice were euthanized, and tumors were excised, photographed, and weighed. Tumor tissues were fixed, embedded in paraffin, and subjected to immunohistochemical staining. Animal experiments were approved by the Animal Ethics Committee of Chongqing Medical University (approval no. 2025 (160)) and followed institutional guidelines and the National Institutes of Health’s Guide for the Care and Use of Laboratory Animals.

### 2.14 Macrophage depletion in the subcutaneous xenograft model

To deplete macrophages *in vivo*, HSC3-shNC and HSC3-shCD93 xenograft-bearing mice were treated with control or clodronate liposomes. A total of 24 male BALB/c nude mice were randomly assigned to four groups: HSC3-shNC + control liposomes, HSC3-shNC + clodronate liposomes, HSC3-shCD93 + control liposomes, and HSC3-shCD93 + clodronate liposomes. Liposomes were administered intraperitoneally at 200 μL per mouse starting 24 h after tumor cell implantation and then every 4 days until the endpoint. Control mice received an equal volume of PBS-loaded control liposomes following the same schedule. For the 2-week xenograft experiment, injections were performed on days 1, 5, 9, and 13 after tumor implantation (Zeisberger et al., 2006; Sharma et al., 2021; Borriello et al., 2022). Tumor volume was monitored with calipers and calculated as length × width^2^/2. Tumors were harvested on day 14, photographed, weighed, fixed, and paraffin-embedded.

F4/80, CD206, CD31, and Ki-67 immunohistochemistry was performed to assess macrophage depletion, M2-like macrophage-associated changes, angiogenesis, and tumor cell proliferation, respectively.

### 2.15 In silico protein–protein docking analysis

The amino acid numbering and domain information of human CD93 were annotated according to UniProt entry Q9NPY3 (The UniProt Consortium, 2025). A structured extracellular construct of CD93 comprising residues 24–430 was used as the receptor for all docking simulations. This region was selected to retain the major folded extracellular portion of CD93 while avoiding the distal flexible extracellular segment. The selection was also supported by available structural studies showing that the N-terminal extracellular domains of CD93 form defined folded modules involved in ligand recognition and receptor organization (Barbera et al., 2023; Xu et al., 2024).

The extracellular domains of Wnt receptor-associated components were prepared from experimentally resolved structures. The LRP6 E1E2 and LRP6 E3E4 domains were obtained from the crystal structures of human LRP6 E1E2 and LRP6 E3E4, respectively, in which these domains were described as rigid extracellular structural blocks of LRP6 (Cheng et al., 2011). The FZD7 cysteine-rich domain (CRD) was extracted from the crystal structure of human FZD7 CRD (Raman et al., 2019).

Protein–protein docking was performed using the ClusPro server, which carries out rigid-body docking, clusters low-energy docked conformations, and ranks representative cluster centers according to weighted energy scores (Kozakov et al., 2017). CD93 residues 24–430 were used as the receptor, whereas LRP6 E1E2, LRP6 E3E4, or FZD7 CRD was used as the ligand. For each docking pair, the top-ranked cluster-center model was selected as the representative docking pose. The number of members in the top-ranked cluster, the cluster-center weighted score, and the lowest-energy score within the corresponding cluster were recorded for comparison (Kozakov et al., 2017). Docking poses were visualized using PyMOL. CD93 was shown in pink, and the corresponding Wnt receptor-associated domain was shown in yellow.

### 2.16 Statistical analysis

Data are presented as mean ± SD unless otherwise indicated. Normality of experimental data was assessed using the Shapiro–Wilk test. Two-group comparisons were performed using an unpaired two-tailed Student’s t-test or Mann–Whitney U test, as appropriate. Multiple-group comparisons were analyzed using one-way ANOVA followed by Tukey’s or Dunnett’s *post hoc* test for normally distributed data, or Kruskal–Wallis test followed by Dunn’s *post hoc* test for non-normally distributed data. For experiments involving two independent variables, two-way ANOVA followed by Sidak’s or Tukey’s multiple-comparison test was used when model assumptions were met; otherwise, data were transformed where appropriate or analyzed by predefined non-parametric simple comparisons. Tumor growth curves were analyzed using two-way repeated-measures ANOVA. For web-based bioinformatics analyses involving multiple testing, platform provided adjusted P values or false discovery rate values. For *in vitro* experiments, n denotes independent biological replicates, and technical replicates, including replicate wells or multiple microscopic fields from the same biological replicate, were averaged before statistical analysis. For *in vivo* studies, n denotes the number of mice per group. Statistical analyses were performed using GraphPad Prism 10.6.0. Significance levels were shown in the figures to preserve readability in complex multi-comparison panels. *P* < 0.05 was considered statistically significant. ns, not significant, *p < 0.05, **p < 0.01, ***p < 0.001, ****p < 0.0001.

## 3 Results

### 3.1 CD93 is upregulated in HNSCC and correlates with poor prognosis and an immunosuppressive microenvironment

**FIGURE 1:**
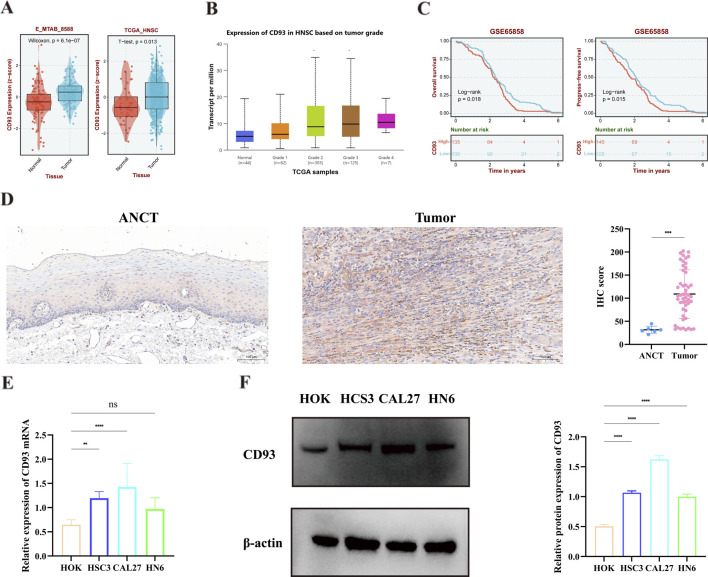
CD93 is upregulated in HNSCC. **(A)** CD93 mRNA levels in HNSCC and normal tissues from TCGA and GEO data. **(B)** Correlation of CD93 levels with histopathological grade in HNSCC. **(C)** Kaplan-Meier analysis of OS and PFS based on CD93 expression in HNSCC from GEO data. **(D)** Representative immunohistochemical staining and quantitative scoring of CD93 in HNSCC (n = 50) and adjacent non-tumor tissues (n = 6). CD93 mRNA **(E)** and protein **(F)** expression in HOK and HNSCC cell lines (HSC3, CAL27, and HN6) (n = 3).

CD93 expression in HNSCC was investigated by analyzing its mRNA levels in the TCGA and GEO databases. Both databases indicated significant overexpression of CD93 in HNSCC compared to normal tissues (*P* < 0.05, Figure 1A). Additionally, increased CD93 expression in HNSCC correlated with advanced tumor grade (Figure 1B) and worse overall and progression-free survival rates (Figure 1C). Subsequently, CD93 expression was assessed in clinical samples, and immunohistochemistry validated higher CD93 protein levels in HNSCC tissues than in neighboring non-tumor tissues (Figure 1D). CD93 levels were higher in HNSCC cell lines compared to HOK cells, with CAL27 and HSC3 cells showing the most significant upregulation in both CD93 mRNA and protein levels (Figures 1E,F).

High CD93 expression in HNSCC tumor tissues was found to have a negative correlation with B cell and CD8^+^ T cell infiltration, while showing a positive correlation with macrophage, CD4^+^ T cell, neutrophil, dendritic cell, and CAF infiltration, along with the immune score (Supplementary Figure S1). This suggests that tumors with high CD93 expression are more likely to reside in an immunosuppressive microenvironment enriched with myeloid and stromal cells but relatively deficient in effector T cells, which may be associated with immune escape and malignant progression. These findings collectively suggest that CD93 is overexpressed in HNSCC and is closely linked to an immunosuppressive TME.

### 3.2 *In vitro* knockdown of CD93 suppresses malignant phenotypes in HNSCC cells

**FIGURE 2:**
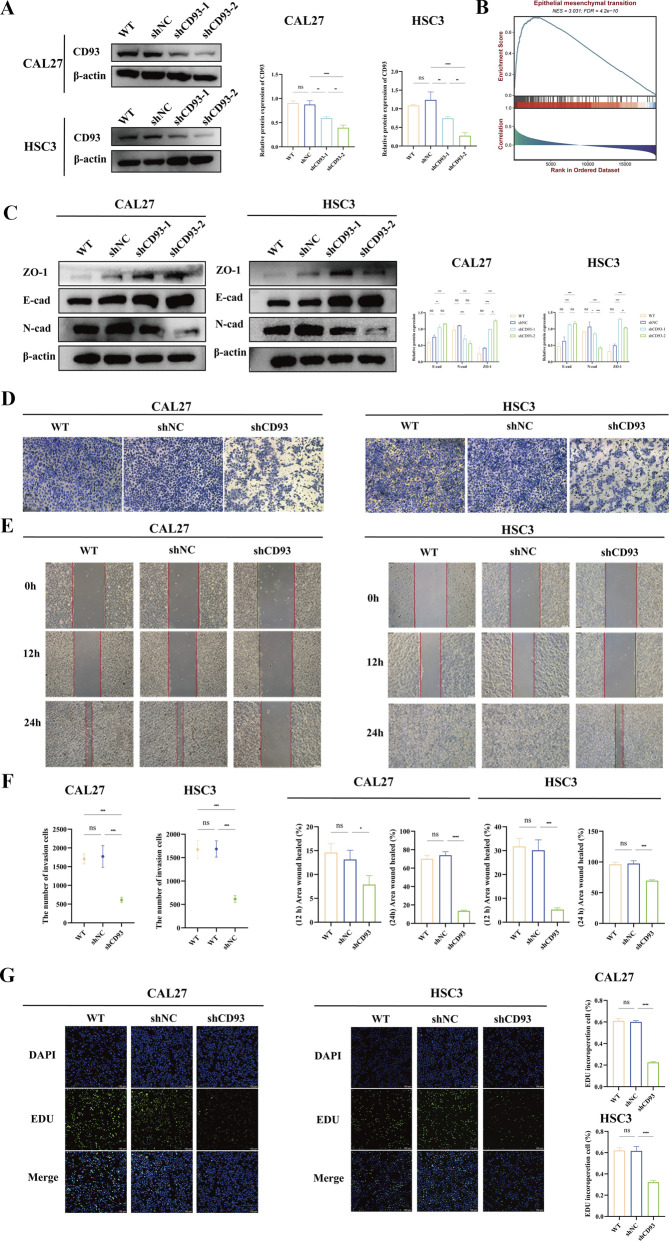
CD93 knockdown attenuates EMT, invasion, migration, and proliferation in HNSCC cells *in vitro*. **(A)** Western blot was used to confirm the efficiency of CD93 knockdown in HSC3 and CAL27 cells. **(B)** GSEA reveals enrichment of the EMT gene set in HNSCC samples with high CD93 expression. **(C)** Western blotting analysis of EMT-related proteins in both cell lines after CD93 knockdown. **(D–G)** Impact of CD93 knockdown on invasion, migration, and proliferation in HSC3 and CAL27 cells. n = 3.

To explore CD93’s role in HNSCC cells, HSC3 and CAL27 cells were selected for functional studies. CD93 knockdown was achieved by shRNA and confirmed by western blotting (Figure 2A). Single-gene GSEA in HNSCC indicated significant enrichment of the EMT gene set in tumors with high CD93 levels, indicating a correlation between elevated CD93 expression and enhanced EMT-associated transcriptional signatures, a tendency toward mesenchymal differentiation, and increased invasiveness (Figure 2B). Consistent with this, CD93 knockdown decreased N-cadherin expression while increasing E-cadherin and ZO-1 levels (Figure 2C). The more effective sequence, shCD93-2, was selected for subsequent experiments. Functionally, knockdown of CD93 obviously inhibited the invasion, migration and proliferation ability in both HSC3 and CAL27 cells (Figures 2D–G). Collectively, these findings suggest that CD93 is associated with the regulation of aggressive phenotypes in HNSCC cells, including EMT, invasion, migration, and proliferation.

### 3.3 CD93 modulates macrophage-associated marker profiles and enhances endothelial tube formation *in vitro*

Given the important roles of tumor-associated macrophages and angiogenesis in HNSCC progression (Troiano et al., 2019; Li et al., 2020; Liu et al., 2024; Alessandrini et al., 2023), we next examined whether CD93-modulated HNSCC cells could influence macrophage-associated marker profiles and endothelial tube formation.

**FIGURE 3:**
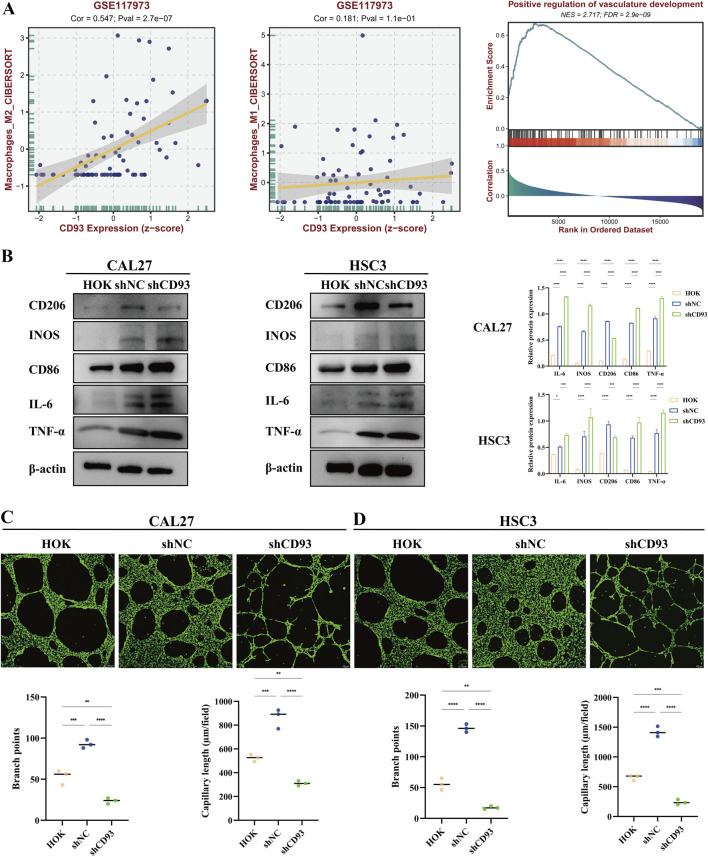
CD93 facilitates HNSCC-induced macrophage M2-like features and endothelial tube formation. **(A)** Bioinformatics-based immune infiltration analysis showing correlations between CD93 expression and estimated infiltration of M2-like macrophages **(a)** and M1-like macrophages **(b)** in HNSCC. GSEA indicates enrichment of the vasculature development gene set in HNSCC samples with high CD93 expression **(c)**. **(B)** Western blotting analysis of M1-like and M2-like markers in THP-1-derived macrophages treated with cell culture supernatant from HOK, CAL27, and HSC3 cells transfected with shNC or shCD93. Tube formation assays of HUVECs treated with cell culture supernatants from CAL27 **(C)** and HSC3 **(D)** cells transfected with shNC and shCD93. n = 3.

Analysis of HNSCC data from the GEO public dataset GSE117973 using the BEST platform revealed a significant correlation between CD93 expression and estimated M2-like macrophage infiltration (*P* < 0.05, Figure 3Aa), whereas no obvious positive trend was observed with estimated M1-like macrophages (*P* > 0.05, Figure 3Ab). In addition, GSEA showed a significant positive enrichment of the positive regulation of angiogenesis gene set in HNSCC, indicating that tumors with high CD93 expression exhibit characteristics that promote angiogenesis (Figure 3Ac).

To evaluate whether CD93 expression in tumor cells was associated with changes in macrophage-related marker expression, THP-1-derived macrophages were exposed to culture supernatants from HOK cells or from CAL27 and HSC3 cells transfected with shNC or shCD93. Compared with HOK-derived supernatant, conditioned media from CAL27 and HSC3 cells increased CD206 expression in THP-1-derived macrophages and also increased iNOS, CD86, IL-6, and TNF-α expression. CD93 knockdown in tumor cells reduced CD206 expression but increased iNOS, CD86, IL-6, and TNF-α expression compared with the shNC group (Figure 3B). These findings suggest that CD93 expression in HNSCC cells contributes to a macrophage-associated marker profile with increased CD206-positive M2-like features.

To explore the impact of CD93 expression on tumor-associated angiogenesis in HNSCC cells, HUVECs were treated with cell culture supernatant from HOK or from CAL27 and HSC3 cells transfected with shNC or shCD93. Compared with HOK, supernatants from CAL27 and HSC3 cells increased both tube formation and branching in HUVECs. CD93 knockdown in tumor cells significantly reduced this tube-forming capacity (*P* < 0.05; Figures 3C,D). These findings indicate that HNSCC cells exert a marked pro-angiogenic effect relative to normal oral epithelial cells and that CD93 contributes substantially to this effect.

### 3.4 CD93-associated wnt/β-catenin signaling promotes malignant phenotypes and tumor cell-mediated macrophage- and endothelial-associated changes *in vitro*

**FIGURE 4:**
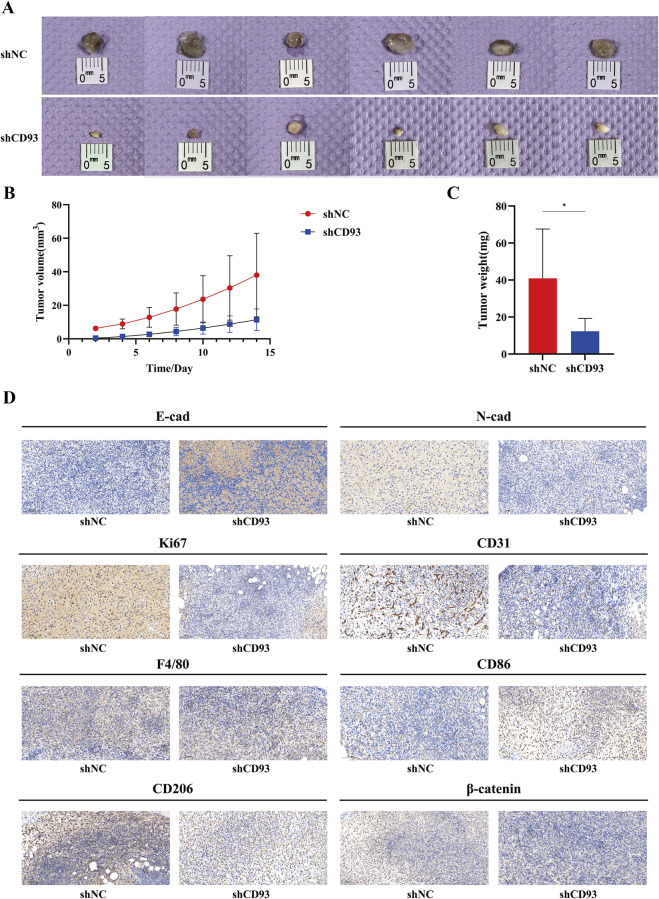
CD93 positively modulates Wnt/β-catenin signaling. **(Aa)** GSEA-Hallmark analysis of TCGA-HNSCC identifying pathways enriched in tumors with high CD93 expression, highlighting Wnt/β-catenin signaling. **(Ab)** GSEA revealed positive enrichment of Wnt/β-catenin signaling in HNSCC samples with high CD93 expression. **(B)** Western blot verifies CD93 overexpression in HSC3 and CAL27 cells. **(C–F)** Western blot analysis of proteins associated with the Wnt/β-catenin pathway following CD93 overexpression in HNSCC cells, combined with treatment with XAV939, or following CD93 knockdown combined with treatment with CHIR99021. **(G,H)** Immunofluorescence staining shows β-catenin nuclear translocation in HNSCC cells with CD93 modulation. n = 3.

Single-gene analysis of CD93 in HNSCC using the BEST platform showed that multiple developmental pathways were enriched in tumors with high CD93 expression, among which Wnt/β-catenin signaling was especially prominent (Figure 4A). We next investigated whether Wnt/β-catenin signaling participates in CD93-associated malignant progression in HNSCC, given the significant impact of the Wnt pathway on tumor invasion, metastasis, and immune microenvironment remodeling in HNSCC (Paluszczak, 2020; Patni et al., 2021).

To explore the impact of CD93 on the canonical Wnt signaling pathway, HSC3 and CAL27 stably overexpressing CD93 were established, confirmed by Western blotting (Figure 4B). CD93 was silenced in CAL27 and HSC3 cells, then treated with the Wnt pathway activator CHIR99021. Conversely, in cells treated with the Wnt pathway inhibitor XAV939, CD93 was overexpressed. Knockdown of CD93 decreased β-catenin expression and its downstream targets, c-Myc and Cyclin D1, which was rescued by CHIR99021. Conversely, CD93 overexpression enhanced these markers, which were suppressed by XAV939 (Figures 4C–F). Immunofluorescence analysis revealed that CD93 knockdown reduced β-catenin nuclear translocation, restored by CHIR99021, while CD93 overexpression promoted β-catenin nuclear translocation, blocked by XAV939 (Figures 4G,H). These findings suggest that CD93 functions as a positive modulator of the canonical Wnt/β-catenin pathway in HNSCC cells.

**FIGURE 5:**
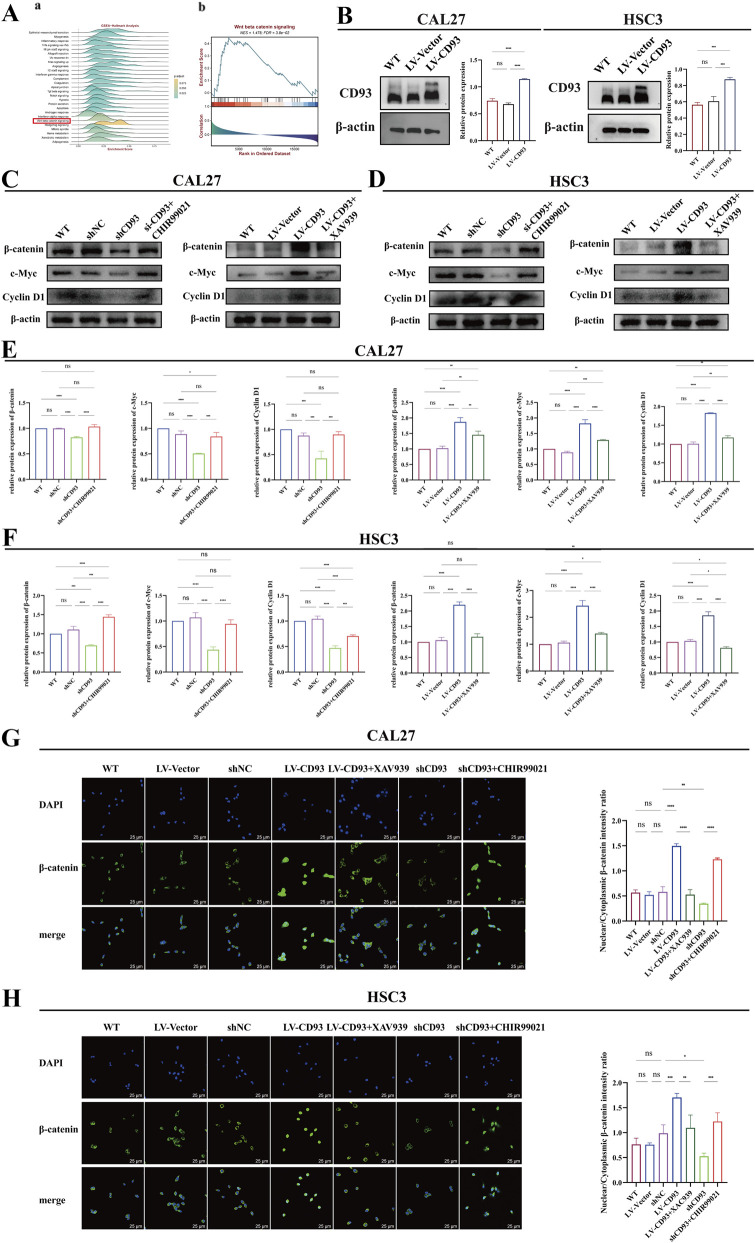
CD93-mediated Wnt/β-catenin signaling promotes HNSCC cells’ invasion and migration. **(A–D)** Transwell invasion assays were conducted to evaluate the effect of CD93 regulation on cell invasion under conditions of Wnt/β-catenin activation or inhibition. **(E–H)** Scratch assays were conducted to evaluate the effect of CD93 regulation on cell migration under conditions of Wnt/β-catenin activation or inhibition. n = 3.

We further investigated whether CD93 modulates tumor cell migration and invasion in HNSCC cells via the Wnt/β-catenin pathway. Results from Transwell and wound-healing assays demonstrated that reducing CD93 levels decreased migration and invasion in both HNSCC cell types, which was reversed by the Wnt pathway activator CHIR99021. In contrast, CD93 overexpression enhanced invasion and migration, whereas the Wnt pathway inhibitor XAV939 partially attenuated these effects (Figures 5A–H). These results collectively suggest that the Wnt/β-catenin signaling pathway at least partially mediates CD93’s role in promoting the malignant phenotype in HNSCC cells.

**FIGURE 6:**
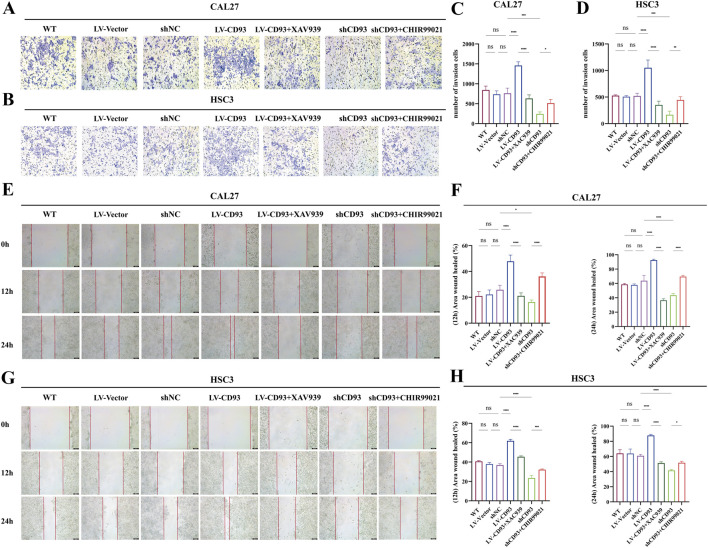
CD93-associated Wnt/β-catenin signaling enhances macrophage-associated M2-like marker profiles and endothelial tube formation. **(A–D)** Western blot analysis of M1-like and M2-like polarization related proteins in macrophages treated with media from CAL27 **(A,C)** and HSC3 **(B,D)**. **(E,F)** *In vitro* endothelial tube formation assay following treatment with media from HNSCC cells. n = 3, *p < 0.05, **p < 0.01, ***p < 0.001.

To explore CD93’s impact on macrophage-associated changes via Wnt/β-catenin signaling in HNSCC cells, macrophage-associated marker profiles were detected. Media from CD93-knockdown HNSCC cells led to decreased CD206 levels and increased M1-like related proteins iNOS, CD86, IL-6, and TNF-α expression, effects reversed by CHIR99021. Conversely, media from CD93-overexpressing HNSCC cells increased CD206 and decreased iNOS, CD86, IL-6, and TNF-α levels, effects attenuated by XAV939 (Figures 6A–D). These findings indicate that elevated CD93 in HNSCC cells is associated with M2-like, tumor-promoting features in macrophages, whereas reduced CD93 is associated with M1-like, inflammation-related features, regulated by the canonical Wnt/β-catenin pathway.

We also examined whether Wnt/β-catenin signaling contributed to the effect of CD93-modulated HNSCC cells on endothelial tube formation. Supernatants from CD93-knockdown HNSCC cells reduced both branch number and tube length in HUVECs, and this reduction was reversed by CHIR99021. In contrast, conditioned medium from CD93-overexpressing HNSCC cells increased branch number and tube length, and these effects were attenuated by XAV939 (Figures 6E,F). These findings suggest that CD93-associated modulation of Wnt/β-catenin signaling in HNSCC cells contributes to enhance the pro-angiogenic activity of tumor cell-derived factors on endothelial cells.

Collectively, these findings suggest that CD93 positively modulates Wnt/β-catenin signaling in HNSCC cells, thereby contributing to enhanced tumor cell invasion and migration, CD206-positive macrophage-associated features, and a pro-angiogenic response in HUVECs.

### 3.5 *In vivo*, CD93 knockdown suppresses xenograft growth

**FIGURE 7:**
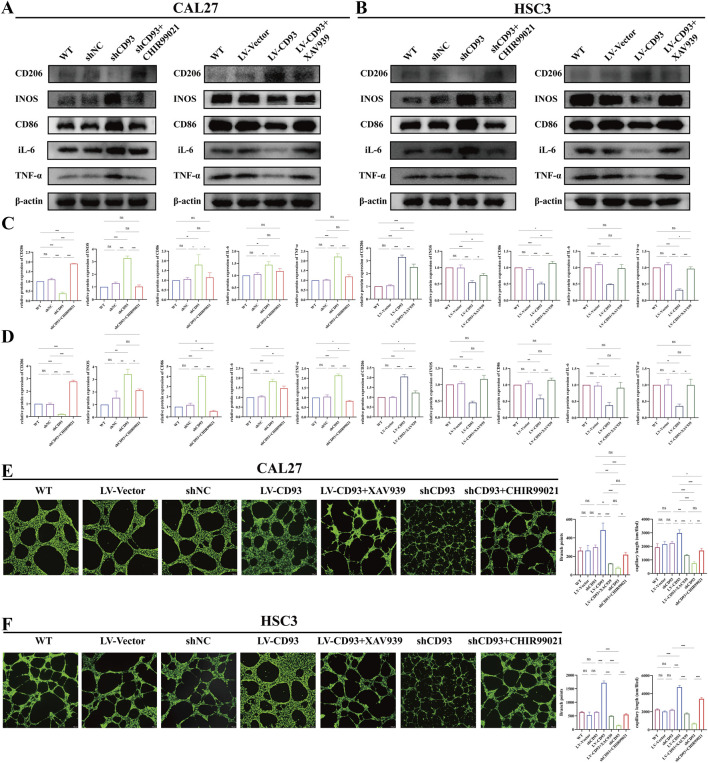
Knocking down CD93 inhibits the growth of xenograft tumors and attenuates EMT, angiogenesis, and M2-like macrophage signals *in vivo*. **(A)** Overall view of subcutaneous xenograft tumors (n = 6) derived from HSC3-shNC and HSC3-shCD93 cells. **(B)** Tumor growth curves of the indicated groups. **(C)** Tumor weight was measured after mice were sacrificed. **(D)** Representative immunohistochemical staining of E-cad, N-cad, Ki67, CD31, F4/80, CD86, CD206, β-catenin in tumor tissue specimens from the HSC3-shNC and HSC3-shCD93 groups. *p < 0.05.

To assess CD93’s role in HNSCC progression *in vivo*, BALB/c nude mice received subcutaneous injections of HSC3 cells with stable shNC or shCD93 expression. After 2 weeks, xenograft tumors from HSC3-shCD93 cells were notably smaller and lighter than those in the HSC3-shNC control group (Figures 7A–C). Immunohistochemistry revealed elevated E-cadherin levels and reduced N-cadherin levels in the shCD93 group, suggesting a mitigation of the EMT phenotype. In parallel, Ki-67 and CD31 expression levels were reduced in tumors with CD93 knockdown, suggesting decreased tumor proliferative activity and angiogenesis. CD93 knockdown reduced expression of β-catenin, suggesting that CD93 may positively regulate the Wnt/β-catenin pathway. Compared with control xenografts, tumors derived from HSC3-shCD93 cells exhibited comparable F4/80 staining, together with reduced CD206 expression and increased CD86 expression, indicating that CD93 knockdown was associated with altered macrophage polarization-related features in the TME (Figure 7D). Taken together, these findings suggest that CD93 knockdown suppresses HNSCC xenograft growth *in vivo*, accompanied by reduced proliferative activity, angiogenesis, EMT-related changes, β-catenin expression, and CD206-positive macrophage-associated signals.

### 3.6 Macrophage depletion attenuates CD93-mediated tumor growth and TME remodeling *in vivo*

**FIGURE 8:**
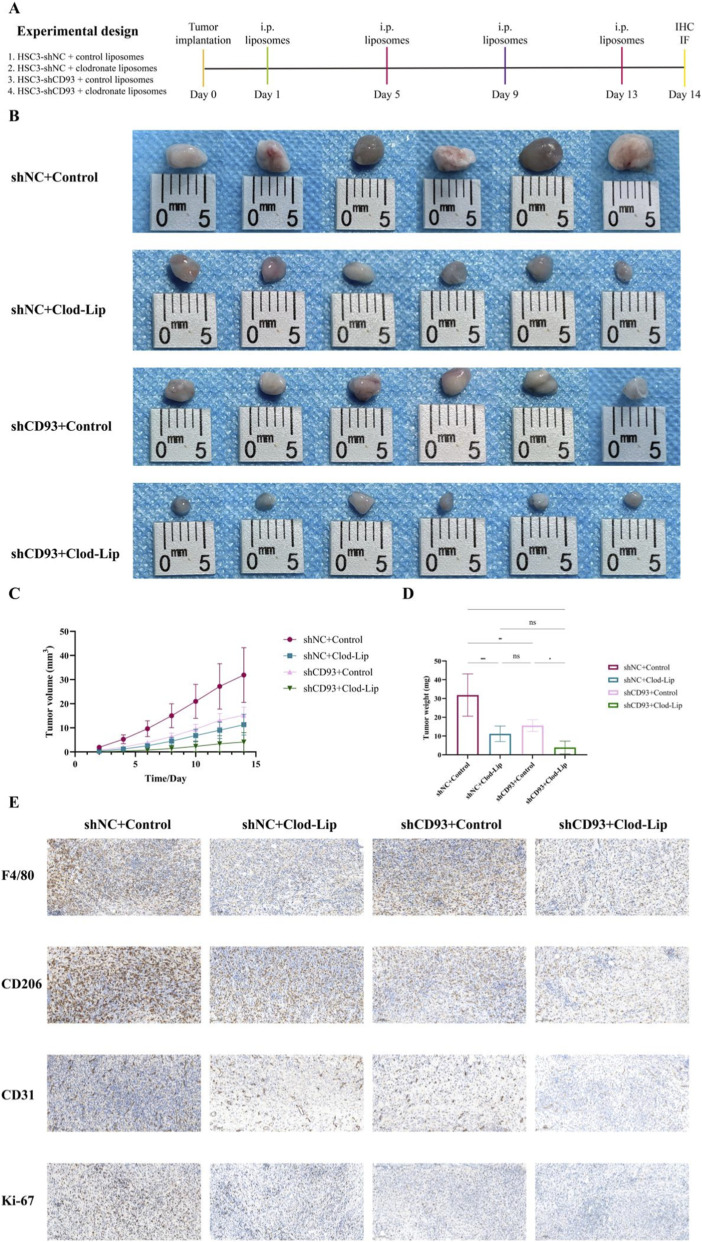
Macrophage depletion attenuates CD93-mediated tumor growth and TME remodeling *in vivo*. **(A)** Schematic representation of the macrophage depletion experiment. HSC3-shNC or HSC3-shCD93 cells were subcutaneously implanted into BALB/c nude mice (n = 6), followed by intraperitoneal administration of control or clodronate liposomes on days 1, 5, 9, and 13 after tumor implantation. Tumors were harvested on day 14. **(B)** Representative images of xenograft tumors from the indicated groups (n = 6). **(C)** Tumor growth curves of the indicated groups. **(D)** Tumor weights at the endpoint. **(E)** Representative immunohistochemical staining of F4/80, CD206, CD31, and Ki-67 in xenograft tumors. F4/80 was used to assess macrophage depletion, CD206 to evaluate M2-like macrophage-associated changes, CD31 to assess angiogenesis, and Ki-67 to evaluate proliferative activity. *P < 0.05, **P < 0.01, ***P < 0.001.

To determine whether macrophages functionally contribute to CD93-mediated tumor growth *in vivo*, we performed macrophage depletion using clodronate liposomes in the HSC3 xenograft model. HSC3-shNC and HSC3-shCD93 cells were subcutaneously injected into BALB/c nude mice, followed by treatment with either control liposomes or clodronate liposomes (Figure 8A). In mice treated with control liposomes, CD93 knockdown significantly suppressed xenograft tumor growth, as reflected by reduced tumor volume and tumor weight (Figure 8B). Notably, clodronate liposome treatment markedly inhibited tumor growth in the HSC3-shNC group, whereas its additional inhibitory effect was less pronounced in the HSC3-shCD93 group. Consequently, macrophage depletion reduced the difference in tumor growth between HSC3-shNC and HSC3-shCD93 tumors, suggesting that macrophages functionally contribute to CD93-mediated tumor progression *in vivo* (Figures 8C,D).

Immunohistochemical analysis confirmed that clodronate liposome treatment markedly reduced F4/80-positive macrophage infiltration in xenograft tumors, demonstrating effective macrophage depletion. In parallel, CD206 staining was decreased after macrophage depletion and was also reduced in tumors with CD93 knockdown, indicating attenuation of CD206-positive macrophage-associated features within the TME. Consistent with the changes in tumor growth, CD31 expression was reduced in both CD93-knockdown tumors and clodronate liposome-treated tumors, suggesting impaired tumor angiogenesis. Ki-67 staining was also decreased, indicating reduced proliferative activity (Figure 8E). Collectively, these results demonstrate that macrophage depletion attenuates CD93-associated tumor growth, angiogenesis, and macrophage-associated microenvironmental changes, supporting the notion that macrophages functionally contribute to CD93-mediated HNSCC progression *in vivo*.

### 3.7 *In silico* docking supports a potential CD93–LRP6 E1E2 spatial association

To further explore whether CD93 may be spatially associated with receptor-proximal components of the Wnt/β-catenin pathway, we performed protein–protein docking analysis using a structured extracellular CD93 construct comprising residues 24–430. Three docking pairs were examined: CD93–LRP6 E1E2, CD93–LRP6 E3E4, and CD93–FZD7 CRD.

**FIGURE 9:**
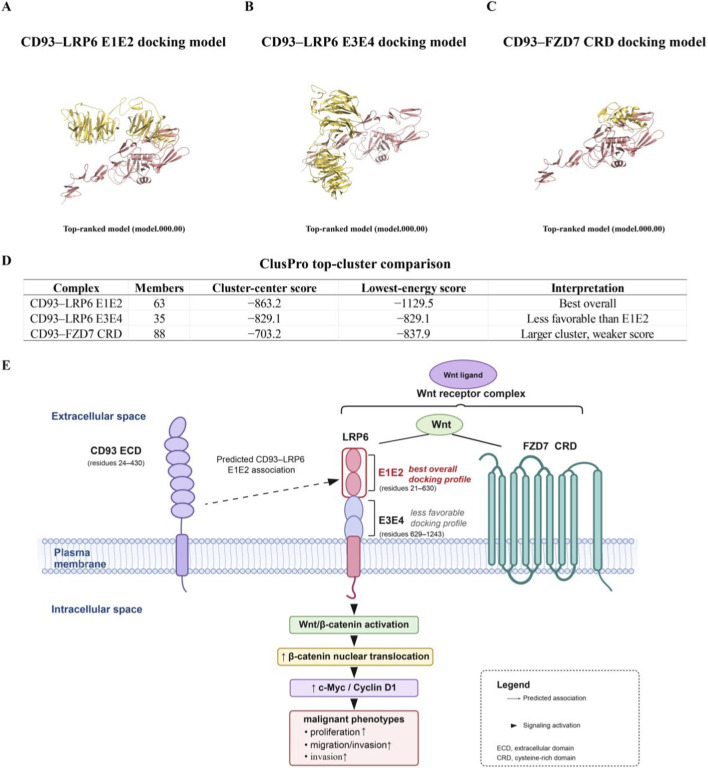
In silico docking analysis of CD93 with receptor-proximal Wnt pathway components. **(A)** Representative top-ranked docking pose of CD93 residues 24–430 with the LRP6 E1E2 extracellular domain. CD93 is shown in pink and LRP6 E1E2 is shown in yellow. **(B)** Representative top-ranked docking pose of CD93 residues 24–430 with the LRP6 E3E4 extracellular domain. **(C)** Representative top-ranked docking pose of CD93 residues 24–430 with the FZD7 cysteine-rich domain (CRD). **(D)** Summary of the top-cluster parameters generated by ClusPro docking analysis. CD93–LRP6 E1E2 showed the most favorable overall docking profile among the tested complexes, with 63 cluster members, a cluster-center weighted score of −863.2, and a lowest-energy score of −1129.5. **(E)** Proposed working model illustrating a potential receptor-proximal association between CD93 and extracellular components of the Wnt receptor complex. The model provides a structural hypothesis for the observed CD93-associated activation of Wnt/β-catenin signaling. These docking results should be interpreted as supportive *in silico* evidence rather than direct biochemical proof of physical binding.

Representative top-ranked docking poses showed that CD93 could form spatially compatible docking conformations with all three extracellular receptor-associated domains (Figures 9A–C). Among the tested combinations, the CD93–LRP6 E1E2 complex showed the most favorable overall docking profile. The top-ranked CD93–LRP6 E1E2 cluster contained 63 members, with a cluster-center weighted score of −863.2 and a lowest-energy score of −1129.5. In comparison, CD93–LRP6 E3E4 showed a top-ranked cluster containing 35 members, with a cluster-center weighted score of −829.1 and a lowest-energy score of −829.1. Although CD93–FZD7 CRD showed the largest top-ranked cluster with 88 members, its cluster-center weighted score and lowest-energy score were less favorable, at −703.2 and −837.9, respectively (Figure 9D).

These results indicate that CD93 displays the strongest predicted spatial compatibility with the LRP6 E1E2 extracellular domain among the tested Wnt receptor-proximal components. Together with the observed CD93-dependent activation of Wnt/β-catenin signaling, these docking data provide supportive *in silico* evidence for a potential receptor-proximal mechanism linking CD93 to canonical Wnt pathway activation (Figure 9E). However, the docking results should be interpreted as predictive structural evidence rather than direct biochemical proof of CD93–LRP6 binding.

## 4 Discussion

**FIGURE 10:**
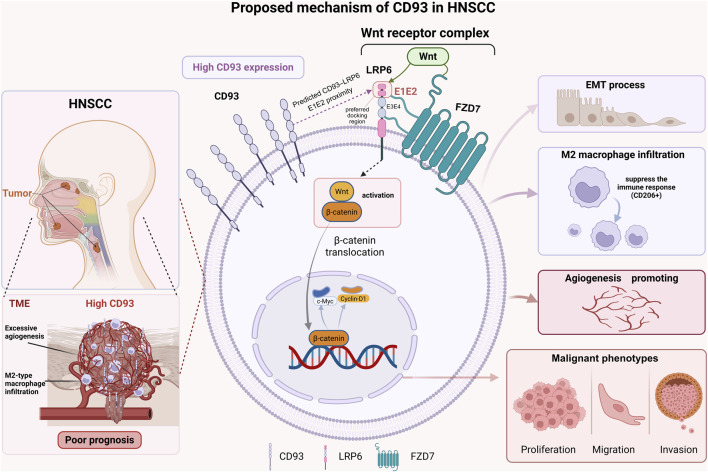
The role of CD93 in promoting the malignant progression of HNSCC. This figure shows a proposed mechanism on the present experimental data and docking analysis. High CD93 expression in HNSCC tumor tissue is proposed to contribute to an aggressive TME characterized by enhanced angiogenesis, M2 macrophage infiltration, and poor prognosis. At the tumor-cell membrane, high CD93 expression may promote canonical Wnt/β-catenin signaling through a receptor-proximal mechanism. Based on molecular docking analysis, the extracellular domain of CD93 is predicted to preferentially approach the E1E2 region of LRP6, which functions together with FZD7 as part of the Wnt receptor complex. This predicted CD93–LRP6 E1E2 proximity may facilitate activation of the FZD7–LRP6 Wnt receptor complex, leading to β-catenin stabilization, β-catenin nuclear translocation, and transcriptional upregulation of downstream targets such as c-Myc and Cyclin D1. Activation of this signaling axis is proposed to promote EMT, M2 macrophage infiltration, angiogenesis, and malignant phenotypes, including proliferation and migration/invasion. This model is based on docking-supported prediction and functional pathway evidence; direct biochemical validation of the CD93–LRP6 interaction is required.

Our study suggests that CD93 is involved in both the aggressive phenotype of HNSCC cells and the remodeling of their surrounding microenvironment. We found that CD93 was upregulated in HNSCC tissues and cell lines, and that higher CD93 expression was associated with advanced tumor grade, unfavorable survival, reduced effector lymphocyte infiltration, and enrichment of myeloid and stromal components. Loss of CD93 suppressed EMT-related changes, migration, invasion, and proliferation, whereas CD93 overexpression produced the opposite effects. These phenotypic changes were accompanied by activation of the Wnt/β-catenin pathway, including increased β-catenin signaling activity and downstream target expression, and were partly reversed by pharmacological inhibition or activation of this pathway. CD93-modulated tumor cells also affected the microenvironment, as shown by altered macrophage-associated marker expression and enhanced endothelial tube formation. *In vivo*, CD93 knockdown reduced tumor growth, Ki-67 expression, CD31-positive angiogenesis, EMT-associated changes, and CD206-positive macrophage-associated signals; macrophage depletion with clodronate liposomes further weakened the growth advantage of CD93-high control tumors. Finally, the docking analysis showed that CD93 had the most favorable predicted spatial compatibility with LRP6 E1E2 among the tested Wnt receptor-proximal components. Taken together, these findings support the mechanism summarized in Figure 10. CD93 may promote HNSCC progression by enhancing Wnt/β-catenin signaling, which in turn strengthens EMT-associated malignant behavior and enables tumor cells to remodel the surrounding microenvironment, as reflected by CD206-positive macrophage-associated changes and increased angiogenic activity. The predicted CD93–LRP6 E1E2 spatial compatibility provides a possible upstream explanation for this Wnt/β-catenin activation, but remains a docking-derived hypothesis that requires biochemical validation (Figure 10).

Rather than acting as a passive background, the TME actively shapes immune escape, angiogenesis, invasion, and treatment resistance in HNSCC (Špiljak et al., 2025). Tumor-associated macrophages are central to this process: recent studies have shown that macrophage–tumor cell crosstalk can promote HNSCC/OSCC progression through inflammatory cytokines, macrophage recruitment, and M2-associated features (Liu et al., 2024; Nie et al., 2024; Sun et al., 2023). This is consistent with our finding that CD93-high tumors were enriched in myeloid and stromal components, whereas CD93 knockdown reduced CD206-positive macrophage-associated signals and weakened the growth advantage of control xenografts after macrophage depletion. In parallel, CD93 promoted EMT-associated changes and endothelial tube formation, while CD93 knockdown reduced CD31-positive angiogenesis *in vivo*, suggesting that EMT-related tumor-cell changes and angiogenic remodeling may be linked rather than separate consequences. Therefore, CD93 may have translational relevance not only as a tumor-associated marker but also as a candidate target for future TME-oriented strategies aimed at reducing macrophage-associated immunosuppression and abnormal angiogenic remodeling. However, this possibility remains to be tested using CD93-targeted interventions and immunocompetent or humanized HNSCC models.

The conditioned-medium model used in this study is a simplified system and does not fully reproduce the complexity of the *in vivo* TME. Macrophage polarization and angiogenesis are also influenced by direct cell–cell contact, extracellular matrix architecture, hypoxia, metabolic stress, and broad cytokine networks. Therefore, our findings mainly indicate that CD93-modulated HNSCC cells can affect macrophage-associated marker expression and endothelial tube formation through soluble factors, whereas the full TME regulatory mechanism requires further validation in more physiologically relevant models.

The Wnt/β-catenin data help explain how CD93 may enhance the aggressive phenotype of HNSCC cells. Aberrant Wnt signaling has been repeatedly implicated in HNSCC and OSCC progression, including EMT, proliferation, invasion, tumor growth, and poor prognosis (Li et al., 2024; Wang et al., 2024; Huang et al., 2024; Xie et al., 2021). In the present study, CD93 knockdown reduced β-catenin, c-Myc, and Cyclin D1 expression and impaired β-catenin nuclear translocation, whereas CD93 overexpression produced the opposite pattern. More importantly, CHIR99021 partially rescued the inhibitory effects caused by CD93 knockdown, while XAV939 attenuated the pro-malignant effects induced by CD93 overexpression. These results support the interpretation that Wnt/β-catenin signaling functionally participates in CD93-associated malignant phenotypes. Nevertheless, the partial rather than complete reversal also indicates that CD93 may not act exclusively through Wnt/β-catenin signaling. Other pathways or tumor-derived mediators may also contribute to the phenotypes observed *in vitro* and *in vivo*.

A key mechanistic issue is whether CD93 directly engages Wnt receptor-proximal components. Our docking analysis was included to address this question at a preliminary structural level, not to claim definitive biochemical binding. LRP6 is a canonical Wnt co-receptor that cooperates with Frizzled receptors during Wnt/β-catenin signal initiation, and structural studies have shown that the extracellular domains of LRP6 participate in ligand- and regulator-related interactions (Cheng et al., 2011; Tsutsumi et al., 2023). Among the tested extracellular components, CD93 showed the most favorable predicted spatial compatibility with LRP6 E1E2. This result is consistent with a receptor-proximal model in which CD93 may facilitate Wnt/β-catenin activation through spatial association with the LRP6 extracellular region. However, protein–protein docking and predicted structures are hypothesis-generating tools (Kozakov et al., 2017; Jumper et al., 2021). They cannot replace biochemical evidence. Therefore, the most appropriate interpretation is that the CD93–LRP6 E1E2 docking result provides a plausible upstream hypothesis for CD93-associated Wnt/β-catenin activation, but direct interaction remains to be validated by co-immunoprecipitation, proximity ligation assay, pull-down assay, surface plasmon resonance, mutational mapping, or related biochemical approaches.

The macrophage-related findings are another important part of the mechanism, especially because HNSCC is frequently characterized by a myeloid-rich and immunosuppressive microenvironment. Recent OSCC work has shown that tumor-cell-conditioned media can drive monocytes toward CD163/CD206-positive immunosuppressive macrophage-like states (Pelaez-Prestel et al., 2024). Similarly, plasma-derived exosomes from HNSCC patients have been reported to induce type 2-like polarization and CXCL4 secretion in monocyte-derived macrophages (Theodoraki et al., 2024). These studies support the biological plausibility of our conditioned-medium experiments. In our system, conditioned media from CD93-high HNSCC cells increased CD206 and reduced iNOS, CD86, IL-6, and TNF-α in THP-1-derived macrophages, whereas CD93 knockdown shifted these markers in the opposite direction. These results suggest that CD93 expression in tumor cells alters macrophage-associated phenotypes through tumor cell-derived soluble factors. However, macrophage states in tumors are highly plastic and cannot be fully defined by a limited M1/M2 marker panel (Liu and Liu, 2024; Murray et al., 2014).

The macrophage depletion experiment provides functional *in vivo* support for the contribution of macrophages to CD93-associated tumor growth. Clodronate liposomes are widely used to deplete phagocytic macrophage populations, and recent nanoplatform studies continue to support their utility for macrophage depletion, although formulation, biodistribution, and cell-targeting specificity remain important considerations (Choi et al., 2024). Classic tumor studies also showed that clodronate-liposome-mediated depletion of tumor-associated macrophages can suppress tumor growth and angiogenesis (Zeisberger et al., 2006). In our xenograft model, clodronate liposomes markedly inhibited tumor growth in HSC3-shNC tumors and narrowed the growth difference between HSC3-shNC and HSC3-shCD93 tumors. This finding suggests that, in the control group with high CD93 expression, macrophages functionally promote tumor growth. Although this model allowed us to evaluate tumor growth, angiogenesis, EMT-associated changes, and macrophage-associated signals, it lacks functional T-cell immunity and therefore cannot fully recapitulate the immune complexity of human HNSCC. Thus, our *in vivo* data support macrophage-associated microenvironmental changes rather than comprehensive antitumor immune remodeling. Future studies using immunocompetent or humanized models are required to define whether CD93 also regulates T-cell-mediated immunity and immunotherapy response.

The angiogenesis-related findings further connect CD93, Wnt/β-catenin signaling, and macrophage-associated TME remodeling. *In vitro*, conditioned media from CD93-overexpressing tumor cells enhanced endothelial tube formation, whereas CD93 knockdown reduced tube formation; these effects were modulated by CHIR99021 and XAV939. *In vivo*, CD93 knockdown decreased CD31-positive angiogenesis, and macrophage depletion further reduced CD31 staining in CD93-high control xenografts. These results are consistent with the established role of CD93 in tumor vascular regulation and with reports that CD93 blockade can normalize tumor vasculature, improve drug delivery, and enhance immune-cell infiltration (Sun et al., 2021; Li et al., 2023). More recent studies also suggest that targeting CD93 on monocytes or blocking the CD93 pathway can enhance CD8^+^ T-cell infiltration and improve antitumor immunity in solid tumors (Jiang et al., 2024; Sun et al., 2025). Although our BALB/c nude mouse model cannot fully evaluate adaptive immune responses, the combined reduction in CD31-positive angiogenesis and CD206-positive macrophage-associated signals supports the concept that CD93 contributes to a pro-angiogenic and macrophage-associated tumor-supportive microenvironment.

From a translational perspective, CD93 may be valuable because it appears to influence several tumor-promoting layers simultaneously: Wnt/β-catenin-associated malignant signaling, EMT-related behavior, angiogenesis, and macrophage-associated TME remodeling. This multi-compartment role may be particularly relevant in HNSCC, where tumor progression and therapeutic resistance are shaped by reciprocal interactions between malignant cells and the surrounding ecosystem (Meng et al., 2024; Gong et al., 2023). However, the therapeutic implication should be stated cautiously. The present study did not test anti-CD93 antibodies, CD93-targeted inhibitors, or combination therapy with immune checkpoint blockade. Moreover, because our *in vivo* experiments were performed in nude mice, the effects of CD93 on CD8^+^ T-cell infiltration, adaptive antitumor immunity, and immunotherapy response remain unresolved in HNSCC.

Our findings support CD93 as a candidate biomarker and mechanistically relevant regulator in HNSCC, but not yet as a clinically validated therapeutic target. CD93 expression may help identify tumors with aggressive phenotypes and macrophage- or angiogenesis-enriched microenvironmental features. Functionally, our loss- and gain-of-function experiments suggest that CD93 contributes to Wnt/β-catenin-associated malignant behavior, endothelial tube formation, and macrophage-associated microenvironmental changes. However, therapeutic feasibility would require direct evidence that pharmacological or antibody-mediated CD93 targeting suppresses HNSCC progression with acceptable efficacy and safety, which was not tested in this study. Previous studies have suggested that blockade of the CD93/IGFBP7 axis can normalize tumor vasculature and improve antitumor therapy responses in solid tumors (Sun et al., 2021; Li et al., 2023; Sun et al., 2025; Qian et al., 2026), supporting the translational interest of CD93. Nevertheless, these strategies remain largely preclinical and have not been validated in HNSCC. Therefore, our data should be interpreted as providing a rationale for future CD93-targeted studies rather than establishing CD93 as an immediately actionable therapeutic target.

This study has several limitations. First, functional validation was mainly performed in CAL27 and HSC3 cells. Although these models showed robust CD93 expression and are widely used HNSCC cell lines, they cannot fully represent the molecular heterogeneity of HNSCC, particularly HPV-positive disease. Future studies should include additional HNSCC models, including HPV-positive cell lines and patient-derived models. Second, although CD93 upregulation was validated in the HNSCC tissue microarray, additional IHC-based correlation analyses between CD93 and EMT, macrophage, angiogenesis, or Wnt pathway markers could not be performed because the tissue sections had been exhausted. Therefore, the clinical association between CD93 and these mechanistic features is currently supported mainly by patient-cohort bioinformatics analyses rather than protein-level spatial validation in patient tissues. Third, immune-infiltration and pathway analyses based on public transcriptomic datasets are indirect and cannot resolve immune-cell localization, functional states, cell–cell interactions, or causal immune remodeling. Therefore, these findings should be considered hypothesis-generating and require validation by multiplex immunofluorescence, flow cytometry, spatial transcriptomics, or single-cell sequencing. Fourth, macrophage-associated changes were evaluated using THP-1-derived macrophages and a limited marker panel. Thus, these data should be interpreted as changes in macrophage-associated marker profiles rather than comprehensive macrophage-state determination. Finally, the docking analysis provides only a preliminary structural hypothesis for possible CD93–LRP6 E1E2 spatial compatibility. Direct biochemical validation, such as co-immunoprecipitation, proximity ligation assay, or surface plasmon resonance, will be required to confirm whether CD93 physically interacts with LRP6 or other Wnt receptor-associated components.

In summary, our findings suggest that CD93 promotes HNSCC progression by enhancing Wnt/β-catenin-related tumor-cell aggressiveness and contributing to macrophage-associated and angiogenic remodeling of the TME. The *in vitro* gain- and loss-of-function experiments support a role for CD93 in EMT, migration, invasion, proliferation, macrophage-associated marker changes, and endothelial tube formation. The xenograft and macrophage depletion experiments further indicate that macrophages functionally contribute to CD93-associated tumor growth *in vivo*. Finally, the docking analysis provides a plausible CD93–LRP6 E1E2 structural hypothesis, but this predicted association requires direct biochemical validation. Together, these data support CD93 as a candidate biomarker and therapeutic target in HNSCC while defining the mechanistic questions that should be addressed in future studies.

## Data Availability

The original contributions presented in the study are included in the article/Supplementary Material, further inquiries can be directed to the corresponding author.
