# Supplementary material for: A preliminary exploration of the role and mechanisms of CD93 in promoting the malignant progression of head and neck squamous cell carcinoma
Source: Front Pharmacol. 2026 Jul 8;17:1847632. doi: 10.3389/fphar.2026.1847632 (PMC13388454; doi:10.3389/fphar.2026.1847632)
Supplement: Supplementary file 1 [file DataSheet1.docx]

**Supplemental Materials**

This supplemental material includes Figure S0 and Figure S1.

| 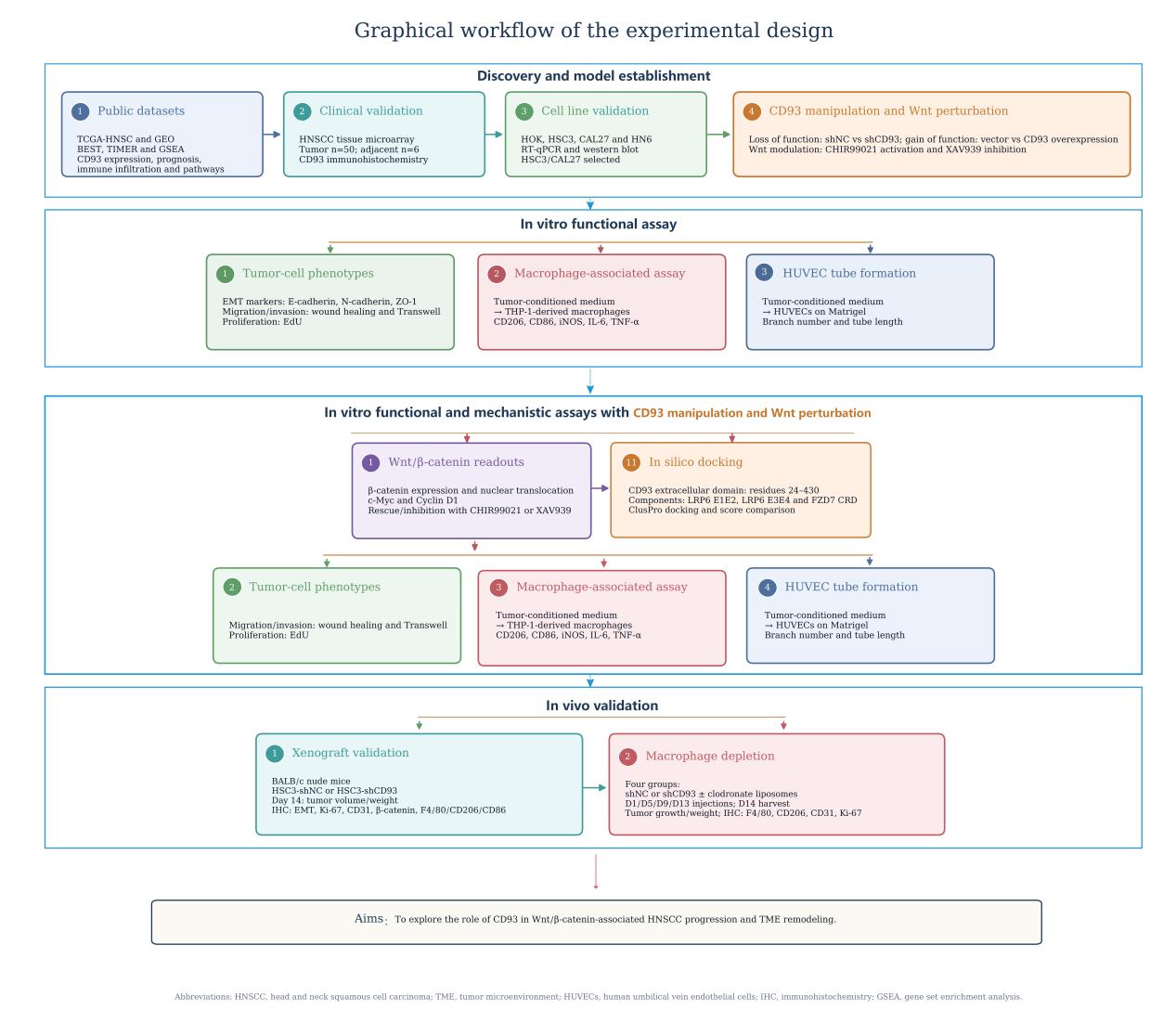 |
| --- |
| **Fig. S0** Graphical workflow of the experimental design |
| The workflow summarizes the major steps of this study, including public database analysis, clinical and cellular validation of CD93 expression, CD93 knockdown and overexpression experiments, Wnt/β-catenin pathway modulation, macrophage- and endothelial-associated assays, xenograft experiments, macrophage depletion, and molecular docking analysis. |

| **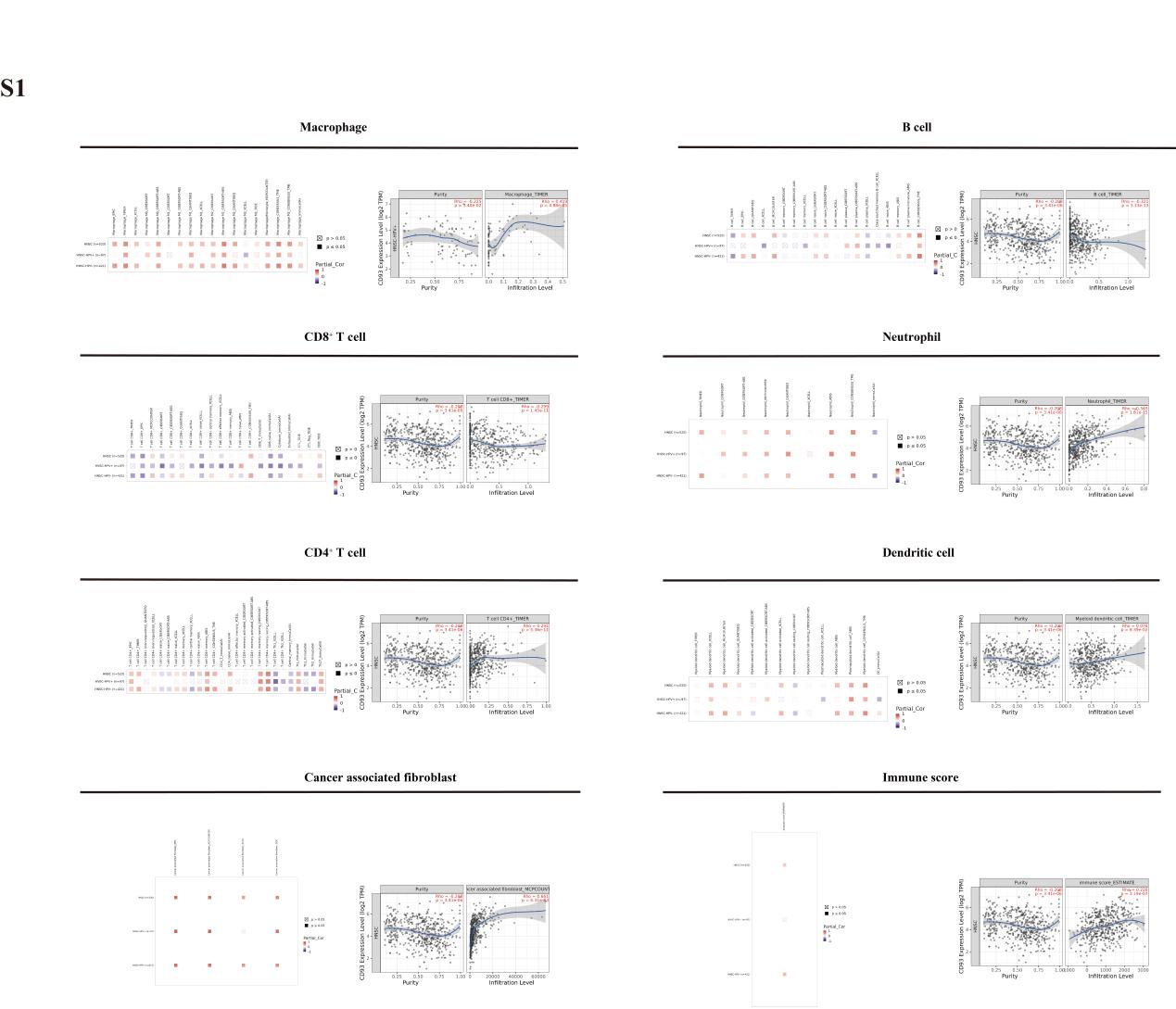** |
| --- |
| **Fig. S1** CD93 is associated with an immunosuppressive tumor microenvironment |
| This figure illustrates the correlation between CD93 expression and the estimated degree of immune cell infiltration in HNSCC. |
